# From sunlight to MS fight: impact of vitamin D levels on multiple sclerosis activity

**DOI:** 10.1007/s10072-025-08729-z

**Published:** 2025-12-23

**Authors:** Marta Vachova, Dominika Stastna, Aneta Mazouchova, Pavla Hruskova, Tomas Uher, Jana Preiningerova Lizrova, Pavel Potuznik, Jiri Drahota, Eva Kubala Havrdova

**Affiliations:** 1 Department of Neurology, Centre of Clinical Neuroscience, First Faculty of Medicine, Charles University in Prague and General University Hospital, Prague, Czechia; 2 Department of Neurology, KZ a.s., Hospital Teplice, Duchcovska 53, Teplice, 415 01 Czechia; 3 ReMuS, The Czech Republic Multiple Sclerosis Patient Registry, Prague, Czechia; 4 Department of Economic Statistics, Prague University of Economics and Business, Prague, Czechia; 5 Department of Clinical Biochemistry and Haematology, KZ a.s., Hospital Teplice, Teplice, Czechia; 6 Department of Neurology, Faculty of Medicine and University Hospital in Pilsen, Charles University, Pilsen, Czechia

**Keywords:** Multiple sclerosis, Vitamin d, Relapse, Cholesterol, Epidemiology, Risk factors

## Abstract

**Background:**

Multiple sclerosis (MS) is a chronic autoimmune disease of the central nervous system with a multifactorial aetiology, including vitamin D levels, that have been linked to disease activity. Given the inconsistent findings on the vitamin D supplementation in MS, we aimed to analyse the connection between 25(OH)D levels and disease activity and to identify an optimal level of 25(OH)D in MS utilizing our real-world database.

**Methods:**

This study utilized a 10-year dataset from the Czech national multiple sclerosis registry (ReMuS), encompassing 1,861 adult MS patients. Patients had a minimum of one year of follow-up, with subgroup analysis for those with at least five years. A mixed-effects model tested the impact of 25(OH)D and cholesterol levels on relapse incidence. Subgroup analysis categorised patients by average 25(OH)D levels and analysed relapse incidence using the Kruskal-Wallis test and Kaplan-Meier survival analysis.

**Results:**

Higher serum 25(OH)D levels and age correlated with reduced relapse risk (*p* < 0.001 for both). Each 10 nmol/L increase in 25(OH)D levels associated with a 6.7% decrease in relapse risk (*p* < 0.001). Cholesterol levels and sex did not significantly affect relapse rate. Subgroup analysis revealed that patients with 25(OH)D levels above 100 nmol/L had significantly fewer relapses compared to those with levels below 75 nmol/L.

**Conclusion:**

Our findings suggest that optimising 25(OH)D levels may reduce the risk of relapse in pwMS. Causation cannot be confirmed. Results highlight the importance of personalized vitamin D supplementation strategies and support the potential benefit of maintaining optimal serum 25(OH)D levels in MS management.

## Highlights

Optimising 25(OH)D levels may reduce multiple sclerosis relapse risk.

Each 10 nmol/L increase in 25(OH)D was linked to a 6.7% reduction in relapse risk.

Patients above 100 nmol/L of 25(OH)D had fewer relapses than those below 75 nmol/L.

No adverse effects were observed in the study.

## Introduction

Multiple sclerosis (MS) is a severe chronic disease of the central nervous system (CNS) and the leading cause of disability among young adults, affecting over 2.8 million people globally [1]. The aetiology of MS is multifaceted, involving genetic, infectious, and environmental risk factors. These include, according to numerous studies, low vitamin D levels, reduced outdoor activity, and genetic polymorphisms that lead to low vitamin D levels. At the same time, the prevalence and incidence of MS show a robust latitudinal gradient, with a strong correlation to UVB exposure that promotes vitamin D synthesis in the skin [2, 3]. Additionally, a higher frequency of late primary Epstein-Barr Virus (EBV) infection at higher latitudes may also contribute to this gradient [4]. Vitamin D might play a role here, enhancing the immune response and increasing CD8 + T cells, potentially mitigating chronic EBV infection [5].

Vitamin D levels are influenced not only by dietary intake, with cholecalciferol from animal sources and ergocalciferol from plant sources, but also by internal production. This internal production relies on cholesterol and the conversion of the precursor 7-dehydrocholesterol in the skin into provitamin D3 through exposure to UVB radiation (290–315 nm). The considered and described effect of cholesterol on the course of autoimmune diseases may thus be partly mediated by this mechanism. Furthermore, the dual sources of vitamin D3 align with its dual roles as both a steroid hormone and a regulator of the immune system, underscoring its importance in immune modulation [3, 6].

The potential benefits of vitamin D supplementation for managing MS have been the focus of numerous studies, though results remain inconclusive. While smaller studies have suggested some positive effects of specific vitamin D doses on MS progression [7–12], evidence from meta-analyses and a few larger trials has been mixed, often limited to analyses of fixed-dose vitamin D regimens [3, 13–17]. The optimal serum concentration of 25(OH)D necessary to achieve maximum health benefits in MS remains undetermined. Current guidelines suggest that individuals with MS aim to maintain serum 25(OH)D levels of at least 75 nmol/L, with an ideal target between 100 and 150 nmol/L, achieved through both dietary intake and adequate sunlight exposure [17].

Given the inconsistent findings on optimal serum 25(OH)D levels in MS, we aimed to address these gaps by utilizing our real-world database from a population commonly affected by vitamin D deficiency, to analyse the connection between 25(OH)D levels and disease activity and to identify an optimal target level of 25(OH)D in MS [18, 19]. Unlike prior studies that have primarily examined fixed-dose vitamin D supplementation, our retrospective study investigates real-world data from a large population cohort with long-term follow-up, where serum 25(OH)D levels are tracked following individually adjusted supplementation dosing, as part of standard clinical practice. Additionally, we choose to consider a role of cholesterol, a precursor in vitamin D synthesis, to understand its potential influence on MS outcomes.

## Methods

### Data collection

The data for this single center retrospective observational cohort study were collected via the Czech national multiple sclerosis registry (ReMuS) between January 2013 and December 2023. Data in ReMuS are collected using the standardised iMed software and undergo a multi-stage quality control process before being released to investigators [20]. ReMuS was approved by The Ethics Committee of The KZ a.s. - Hospital Teplice. Informed consent was obtained from all patients enrolled in ReMuS and includes approval for retrospective data analysis. All relevant operational and legal information can be obtained via the ReMuS website (www.multiplesclerosis.cz).

### Population of interest and variables assessed

The analysis had two parts. The main analysis focused on adult MS patients with at least a year of follow-up, and the subgroup analysis included patients with at least five years of follow-up (in case of longer follow-up, the last five years were evaluated). In both analyses, we included only patients with at least two visits per year frequency (including laboratory tests), with no biotin supplementation. Age, sex, MS duration, incidence of relapses, Expanded Disability Status Scale (EDSS; assessed by EDSS certification holders), total cholesterol, 25(OH)D levels, and information on biotin use were evaluated (the use of biotin-streptavidin binding in immunochemical reactions during vitamin D concentration assessment may affect results in patients taking biotin supplements [21]). The potential occurrence of adverse events in association with elevated 25(OH)D levels is assessed twice a year during regular visits. Vitamin D supplementation was not restricted in any way and depended on the detected 25(OH)D level and the assessment of the treating physician. Additionally, in a randomly selected 10% subsample of the patients with at least five years of follow-up, we evaluated magnetic resonance imaging (MRI) activity. For each patient, we identified MRI scans performed closest to baseline + 2 years and baseline + 5 years. Patients without available MRI data within one year of these time points were excluded from this subanalysis. MRI activity was defined as the presence of new or enlarging T2 lesions between these two scans, based on assessment by a board-certified radiologist.

### Vitamin D evaluation

As the main way of assessing vitamin D status, we choose the levels of 25(OH)D, along with its biologically inactive precursors, 25-hydroxyvitamin D3 and 25-hydroxyvitamin D2, which are products of the initial hydroxylation of exogenous and endogenous vitamin D. Subsequent hydroxylation of 25(OH)D results in 1,25-dihydroxyvitamin D (when calcium utilisation is required), or the biologically less effective 24,25-dihydroxyvitamin D. Measuring these active forms is less suitable for our analysis due to their dependence on calcium metabolism.

25(OH)D levels were measured twice a year, six months apart, as part of standard clinical care at MS centres. This biannual sampling takes into account possible seasonal variations in vitamin D levels throughout the year. The laboratory used the same methods and analyser over the selected study period. 25(OH)D concentrations were measured by competitive electrochemiluminescence analysis, with standardisation adhering to ID-LC-MS/MS and NIST 2972 standard reference material. The assay is also designed to specifically block cross-reactivity with 24,25-dihydroxyvitamin D by using a monoclonal antibody, which ensures accurate measurement of 25(OH)D, as a primary marker of vitamin D metabolism. The measuring range of the method is 7.5*–*300 nmol/L.

### Statistical analyses

Within the main analysis, we first tested the hypothesis that 25(OH)D and cholesterol levels influence the likelihood of relapse development. The occurrence of a relapse within the ensuing year served as the dependent dichotomous variable. We utilised mixed-effects models to accommodate multiple observations from individual patients, which is denoted in regression notation as (1 | Patient´s ID). This approach essentially mirrors logistic regression, allowing us to assess repeated measures in individual patients and account for patient-specific variations over time. Laboratory measurements of 25(OH)D and cholesterol levels were treated as independent variables. The effects of these variables were adjusted for sex, age, and the Expanded Disability Status Scale (EDSS) to control for confounding factors. The logistic regression model was structured as follows: Relaps in the following 12 months ~ 1 + Vitamin D + Sex + Age + Cholesterol + log(EDSS + 1) + (1 | Patient´s ID). This model specification allows us to assess the fixed effects of 25(OH)D and cholesterol on the probability of relapse while adjusting for relevant demographic and clinical variables.

Second, within a subgroup analysis, to validate the relationship between 25(OH)D levels and the risk of relapse, we employed a distinct methodological approach using the cohort with at least five years of follow-up. Mean 25(OH)D levels were calculated based on measurements taken during the first two years of the evaluated five-year period. Based on these 25(OH)D levels, patients were categorised into three groups:

- Group A: patients with an average 25(OH)D level below 75 nmol/L.
- Group B: patients with an average level between 75 nmol/L and 100 nmol/L.
- Group C: patients with an average level above 100 nmol/L.

**Fig. 1:**
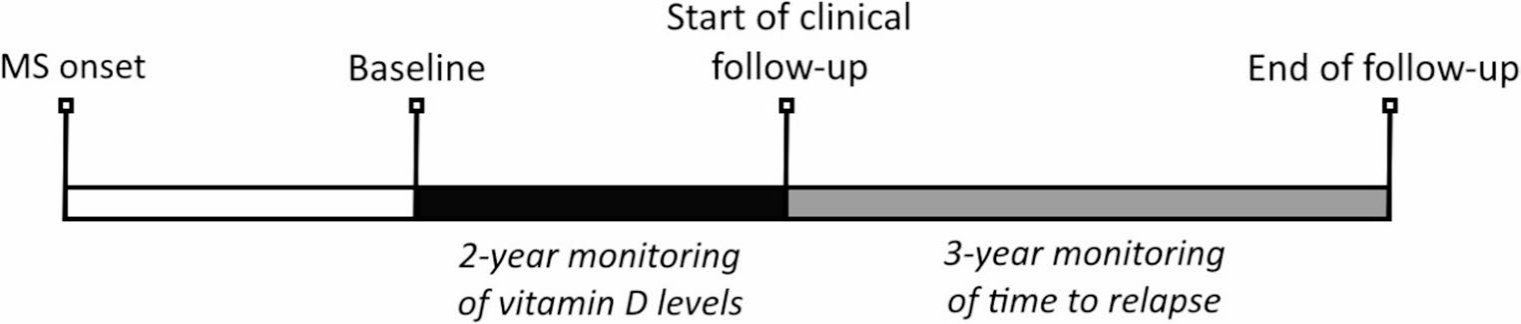
Subgroup analysis design

The subsequent three years of follow-up data were used to assess the occurrence of relapses (Fig. 1). To analyse the impact of 25(OH)D levels on relapse incidence, we employed the Kruskal-Wallis test to evaluate the dependency across the groups. Furthermore, Kaplan-Meier survival analysis was used to compare the time to relapse among the three groups. The proportion of a randomly selected subsample of patients with MRI activity (defined as the presence of new or enlarging T2 lesions) was compared between the three vitamin D groups using Fisher’s exact test.

Cohort characteristics were summarised using mean (SD), median, minimum and maximum, and ranges as appropriate. P-values less than 0.05 (two-tailed) were considered significant. Statistical analyses were done in R version 4.1.2.

### Data Availability

Essential data relevant to the study are included in the article. The data underlying this article are stored in a protected server environment managed by the ReMuS registry and cannot be shared publicly due to data protection regulations. Access to the data is granted to authorised researchers after they have made a request to the ReMuS Scientific Board and the Registry Management Board, provided that their request complies with ethical standards and data protection laws.

## Results

### The effect of selected variables on the occurrence of relapse within one year

The main analysis model included 1,861 patients and 9,865 observations. 25(OH)D values ranged from 7.5 to 296 nmol/L (mean 87.5 ± 31.6), and cholesterol from 1.68 to 10.4 nmol/L (mean 5.0 ± 1.0). 25(OH)D levels above 300 nmol/L and zero values were discarded from the analysis. No adverse effects of elevated 25(OH)D levels were observed during follow-up.

A model examining the relationship between 25(OH)D levels, sex, age, cholesterol levels and EDSS (independent variables) and the occurrence of relapses within the following year (dependent variable) explained 71.5% of the variability in relapse occurrence, with a marginal R² of 19.8%. 25(OH)D levels (p-value < 0.001), age (p-value < 0.001), and the EDSS score (p-value < 0.001) were associated with occurrence of relapsing activity. Specifically, for every 10-unit increase in 25(OH)D, there was a 6.7% reduction in the risk of relapse. Similarly, each additional year of patient age was associated with a 12.4% decrease in relapse risk. Conversely, cholesterol levels and sex did not show an impact on relapsing activity within a 12-month period (with p-values of 0.262 and 0.529, respectively; Table 1).

**Table 1:** The effect of selected variables on the occurrence of relapse within one year (fixed effects parameter estimates)

Parameter	Estimate	SE	exp(B)	95% confidence interval (exp(B))	z-score	*p*-value
Intercept	−2.630	0.108	0.072	0.058–0.089	−24.285	< 0.001
Vitamin D	−0.007	0.002	0.993	0.990–0.997	−3.876	< 0.001
Sex	−0.102	0.161	0.903	0.658–1.240	−0.629	0.529
Age	−0.132	0.008	0.877	0.863–0.891	−16.080	< 0.001
Cholesterol	0.058	0.052	1.060	0.958–1.172	1.121	0.262
Log(EDSS + 1)	3.740	0.495	42.087	15.963–110.963	7.561	< 0.001

### Subgroup analysis of patients with different 25(OH)D levels

Subgroup analysis was performed by stratifying patients into three groups based on their 25(OH)D levels. The characteristics of the whole cohort and groups A, B, and C (with median 25(OH)D levels 62,7, 86,7 and 114,7 nmol/L respectively), are summarised in Table 2. There were no significant differences between the groups in their characteristics, such as disease duration or EDSS, except for age and 25(OH)D levels. Also, an evaluation of specific medications within DMT groups showed no significant result. The incidence of relapses was assessed between 3 and 5 years after baseline. We observed a total of 316 relapses, an average of 0.36 relapses per patient over three years of follow-up (annualised relapse rate of 0.12). In group A (*n* = 276), there were a total of 119 relapses (i.e. 0.43 relapses per patient, with 67% of patients in group A being relapse-free). In group B (*n* = 312), there were a total of 115 relapses (i.e. 0.37 relapses per patient, with 71% being relapse-free). In group C (*n* = 284) with the highest vitamin D levels, there were a total of 82 relapses (i.e. 0.29 relapses per patient, with 77% of patients being relapse-free) (Table 3).

**Table 2:** The characteristics of the subgroup analysis cohort

CHARACTERISTIC	GROUPS BY MEAN 25(OH)D AT BASELINE (NMOL/L)				
	Overall *N* = 872	A (0–75) *N* = 275	B (75–100) *N* = 312	C (100+) *N* = 285	*p*-value^1^
25(OH)D levels Mean (median)	88,9 (87,0)	60,1 (62,7)	87,0 (86,7)	118,9 (114,7)	NA
Sex (female, %)	644 (74%)	202 (73%)	232 (74%)	210 (74%)	> 0.9
Age at Baseline, mean (SD)	43.3 (12.0)	40.3 (11.4)	43.8 (11.5)	45.5 (12.6)	< 0.001
Disease duration at Baseline years, mean (SD)	11 (8.9)	10.8 (8.1)	11.0 (8.3)	11.3 (10.1)	0.6
EDSS at Baseline, median (range)	3.0 (0.0, 9.0)	3.0 (1.0, 9.0)	2.5 (1.0, 8.0)	3.0 (0.0, 8.0)	0.7
DMT at start of clinical follow up					0.3
HE-DMT	249 (29%)	90 (33%)	85 (27%)	74 (26%)	
P-DMT	407 (47%)	126 (46%)	143 (46%)	138 (48%)	
No DMT	216 (25%)	59 (21%)	84 (27%)	73 (26%)

**Table 3:** Incidence of relapses in groups based on baseline 25(OH)D levels

NO. OF RELAPSES	Overall *N* = 872	Group A (0–75 nmol/L) *N* = 276	Group B (75–100 nmol/L) *N* = 312	Group C (> 100 nmol/L *N* = 284
0	625 (72%)	186 (67%)	220 (71%)	219 (77%)
1	183 (21%)	66 (24%)	69 (22%)	48 (17%)
2	53 (6.1%)	19 (6.9%)	20 (6.4%)	14 (4.9%)
3	9 (1.0%)	5 (1.8%)	2 (0.6%)	2 (0.7%)
Mean	0.362	0.431	0.369	0.289
ARR	0.121	0.144	0.123	0.096

**Fig. 2:**
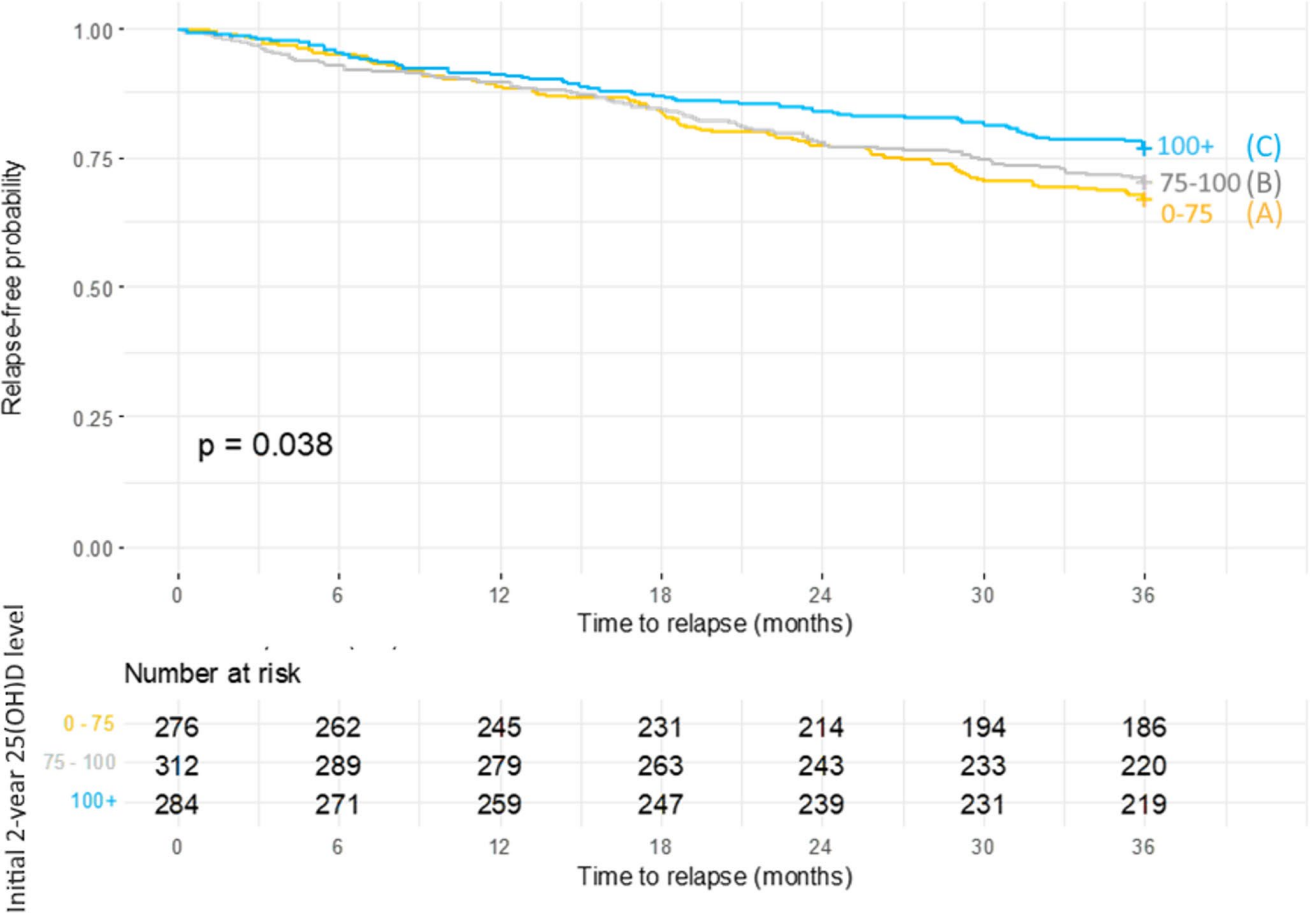
Time to relapse over three years of follow up

The Kaplan-Meier analysis revealed a statistically significant difference in the time to relapse across the groups (p-value 0.038). Subsequent post hoc testing aimed to determine which specific groups differed. The results showed no statistically significant differences between Group A and Group B (p-value of 0.457), nor between Group B and Group C (p-value of 0.100). However, a significant difference was found between Group A and Group C (p-value 0.035), indicating that patients with 25(OH)D levels above 100 nmol/L experience significantly lower frequency of relapses than patients with 25(OH)D levels below 75 nmo/L (Fig. 2).

Brain MRI data were available for 84 of 89 patients (94.4%) in the randomly selected subsample. The overall rate of MRI activity was 11.9% (10/84). MRI activity was observed in 4/28 patients (14.3%) in Group A, 3/29 patients (10.3%) in Group B, and 3/27 patients (11.1%) in Group C. These differences were not statistically significant.

## Discussion

This large retrospective observational study of 1861 people with MS, utilizing data from the Czech national multiple sclerosis registry (ReMuS) showed a significant association between higher 25(OH)D levels and a reduced risk of relapse. Specifically, the logistic regression analysis demonstrated that for every 10-unit increase in 25(OH)D levels, there was a 6.7% decrease in relapse probability, while cholesterol levels (as a vitamin D precursor) was not found to influence relapse rates significantly. Subgroup analysis further corroborated these findings, showing that patients with average 25(OH)D levels above 100 nmol/L had fewer relapses and longer time to relapse over the three years of follow up compared to those with vitamin D insufficiency (defined as levels below 75 nmol/L), highlighting the potential protective effect of higher 25(OH)D concentrations.

The role of vitamin D in multiple sclerosis (MS) has been extensively studied. Observational studies including ours suggest a correlation between low vitamin D levels and increased MS activity, these do not necessarily prove causation.

Our study contributes to the existing body of knowledge on vitamin D in MS by providing data from a large, homogeneous cohort of Caucasians at a latitude where this issue has not been extensively studied. Additionally, our study involved longer clinical monitoring to assess relapse rate compared to previous studies. This research can be extended to other European countries with similar populations. Low vitamin D levels in populations around the 50th parallel remain a major concern [18, 19]. The limitations of this study include the absence of a control group, the omission of some co-factors (e.g. comorbidities) that may also influence disease activity, and the lack of MRI data. Although we performed an exploratory MRI subanalysis in a randomly selected subset of 84 patients, its interpretability is limited by the small sample size. While the trend in MRI activity numerically mirrored the clinical relapse data, the differences were not statistically significant and the results should be considered hypothesis-generating only. Our model explained 71.5% of the variance in disease activity, indicating that other factors might also play a role.

Our results are consistent with findings from other studies. A study involving 74 pwMS in Asia also showed a significantly lower incidence of relapses over a two-year period in patients with higher 25(OH)D levels (45.53 ± 10.52 nmol/L vs. 73.01 ± 14.30 nmol/L) [22]. Several other observational studies have reported a reduction in the risk of subsequent relapses by 14–34% for each 25 nmol/L increase in 25(OH)D levels. However, these studies were limited by small samples (73–469 pwMS) and were conducted in different geographical regions, particularly with regard to latitude [8, 11, 12]. On the other hand, some smaller studies have indicated that the effect of vitamin D on relapse rates may be confined only to specific populations of pwMS, such as those with young age [10] or certain genetic risk-allele constitutions [9]. Notably, the largest study to date, which included 1,482 patients, also found no significant association between 25(OH)D levels and relapse rates. However, this study did find a difference when comparing activity on MRI, which is considered a more sensitive parameter of inflammatory activity [23].

While our study does not establish a causal relationship between low vitamin D levels and relapse rates, there is substantial evidence supporting the potential benefits of vitamin D supplementation in managing autoimmune disorders. Several randomized clinical trials have shown positive effects of vitamin D supplementation, such as a reduction in inflammatory markers and disease activity in systemic lupus erythematosus with 2000 IU/day of vitamin D [24] and improved immune regulation and reduced thyroid autoantibodies in Hashimoto’s thyroiditis [25]. In a study of 30 patients with clinically isolated syndrome (CIS), as an early stage of MS, with low serum 25(OH)D levels, weekly administration of 50,000 international units (IU) of vitamin D3 for 12 months significantly reduced the risk of developing definite MS by 68.4% and lowered the incidence of brain MRI lesions compared to a placebo [15].

However, according to a meta-analysis of nine studies, both low- and high-dose supplementation with vitamin D had no effect on relapse rates compared with placebo [16]. This was also concluded by a recent double-blind trial in Australia and New Zealand where 199 patients were randomised to receive either a placebo, 1,000 IU, 5,000 IU, or 10,000 IU of oral vitamin D3 daily for up to 48 weeks. The results indicated that vitamin D3 supplementation had no effect on the reduction of MS activity in patients with high-risk CIS [13]. The primary limitation of this study is that some patients were not vitamin D deficient at the outset, while others did not achieve optimal levels during the study, potentially affecting the overall outcomes. This underscores the need for individualized dosing rather than a one-size-fits-all strategy.

Our dataset provides valuable insights into vitamin D levels within a population where a standard recommendation of supplementing a minimum of 2000 IU per day is integrated into routine medical care. Vitamin D levels were regularly monitored at least twice a year to accommodate for seasonal fluctuations and doses are adjusted to achieve a target of 75 nmol/L and not withdrawing the vitamin D supplementation until reaching 150 nmol/L. Despite this approach, real-world evidence from our study shows that a significant portion of the population fails to reach vitamin D levels above 75 nmol/L, and these individuals experience more relapses.

In our study, we choose to evaluate actual serum levels rather than focusing on prescribed dose of vitamin D. This method is partly shared with a Danish prospective cohort study of 170 patients, where supplementation was given at levels below 50 mmol/L. Correction of hypovitaminosis D in clinical practice by recommending oral D3 supplements resulted in increases in 25(OH)D levels in serum, which were associated with decreases in ARR in pwMS [7].

Given that vitamin D supplementation is a safe and inexpensive treatment modality, it is important to continue to explore its potential in managing multiple sclerosis. Personalized dosing seems to be a critical factor in further vitamin D studies in MS, although challenges remain, such as unknown optimal serum levels for specific clinical situations, and barriers of reaching them, such as non-compliance, genetic variability affecting vitamin D metabolism, or increased consumption during inflammation, where low vitamin D levels might be a consequence rather than a cause.

## Conclusion

Our study shows that increasing 25(OH) levels may reduce the risk of relapse in pwMS in central Europe, regardless of the type of DMT. Specifically, every 10 nmol/L increase in vitamin D seems to correlate with a 6.4% reduction in relapse risk. Patients with 25(OH)D levels above 100 nmol/L experienced significantly fewer relapses compared to those below 75 nmol/L, with no adverse effects observed. These findings align with previous studies and suggest vitamin D supplementation as a safe and cost-effective intervention. We recommend maintaining serum 25(OH)D levels within 100–150 nmol/L to potentially enhance MS management alongside all existing therapies. However, further research, particularly randomised controlled trials, is needed to confirm the efficacy of targeted 25(OH)D supplementation.

## References

[1] Walton C, King R, Rechtman L (2020) Rising prevalence of multiple sclerosis worldwide: insights from the Atlas of MS, third edition. Mult Scler J 26:1816–1821. 10.1177/1352458520970841 10.1177/1352458520970841 PMC7720355 33174475

[2] Simpson S, Wang W, Otahal P et al (2019) Latitude continues to be significantly associated with the prevalence of multiple sclerosis: an updated meta-analysis. J Neurol Neurosurg Psychiatry 90:1193–1200. 10.1136/jnnp-2018-320189 31217172 10.1136/jnnp-2018-320189

[3] Sintzel MB, Rametta M, Reder AT (2018) Vitamin D and multiple sclerosis: a comprehensive review. Neurol Ther 7:59–85. 10.1007/s40120-017-0086-4 29243029 10.1007/s40120-017-0086-4 PMC5990512

[4] Pender MP (2012) <article-title update="added">CD8+ T-cell deficiency, Epstein-Barr virus infection, vitamin D deficiency, and steps to autoimmunity: a unifying hypothesis. Autoimmune Dis 2012:189096. 10.1155/2012/189096 22312480 10.1155/2012/189096 PMC3270541

[5] Houen G, Trier NH (2021) Epstein-Barr virus and systemic autoimmune diseases. Front Immunol 11:587380. 10.3389/fimmu.2020.587380 33488588 10.3389/fimmu.2020.587380 PMC7817975

[6] Lorincz B, Jury EC, Vrablik M (2022) The role of cholesterol metabolism in multiple sclerosis: from molecular pathophysiology to radiological and clinical disease activity. Autoimmun Rev 21:103088. 10.1016/j.autrev.2022.103088 35398271 10.1016/j.autrev.2022.103088

[7] Laursen JH, Søndergaard HB, Sørensen PS (2016) Vitamin D supplementation reduces relapse rate in relapsing-remitting multiple sclerosis patients treated with natalizumab. Mult Scler Relat Disord 10:169–173. 10.1016/j.msard.2016.10.005 27919484 10.1016/j.msard.2016.10.005

[8] Mowry EM, Krupp LB, Milazzo M et al (2010) <article-title update="added">Vitamin D status is associated with relapse rate in pediatric‐onset multiple sclerosis. Ann Neurol 67:618–624. 10.1002/ana.21972 20437559 10.1002/ana.21972

[9] Mowry EM, Waubant E, McCulloch CE et al (2012) Vitamin D status predicts new brain magnetic resonance imaging activity in multiple sclerosis. Ann Neurol 72:234–240. 10.1002/ana.23591 22926855 10.1002/ana.23591 PMC3430977

[10] Muris AH, Smolders J, Rolf L et al (2016) Vitamin D status does not affect disability progression of patients with multiple sclerosis over three-year follow-up. PLoS One. 10.1371/journal.pone.0156122 27276080 10.1371/journal.pone.0156122 PMC4898831

[11] Runia TF, Hop WCJ, De Rijke YB et al (2012) Lower serum vitamin D levels are associated with a higher relapse risk in multiple sclerosis. Neurology 79:261–266. 10.1212/WNL.0b013e31825fdec7 22700811 10.1212/WNL.0b013e31825fdec7

[12] Simpson S, Taylor B, Blizzard L et al (2010) Higher 25-hydroxyvitamin D is associated with lower relapse risk in multiple sclerosis. Ann Neurol 68:193–203. 10.1002/ana.22043 20695012 10.1002/ana.22043

[13] Butzkueven H, Ponsonby AL, Stein MS et al (2024) Vitamin D did not reduce multiple sclerosis disease activity after a clinically isolated syndrome. Brain 147:1206–1215. 10.1093/brain/awad409 38085047 10.1093/brain/awad409 PMC10994527

[14] Cassard SD, Fitzgerald KC, Qian P et al (2023) High-dose vitamin D3 supplementation in relapsing-remitting multiple sclerosis: a randomised clinical trial. EClinMed 59:101957. 10.1016/j.eclinm.2023.101957 10.1016/j.eclinm.2023.101957 PMC10130605 37125397

[15] Derakhshandi H, Etemadifar M, Feizi A et al (2013) Preventive effect of vitamin D3 supplementation on conversion of optic neuritis to clinically definite multiple sclerosis: a double-blind, randomized, placebo-controlled pilot clinical trial. Acta Neurol Belg 113:257–263. 10.1007/s13760-012-0166-2 23250818 10.1007/s13760-012-0166-2

[16] Hanaei S, Sahraian MA, Mohammadifar M et al (2021) Effect of vitamin D supplements on relapse rate and expanded disability status scale (EDSS) in multiple sclerosis (MS): a systematic review and meta-analysis. Int J Prev Med 12:42. 10.4103/ijpvm.ijpvm_208_20 34211673 10.4103/ijpvm.IJPVM_208_20 PMC8223916

[17] Sîrbe C, Rednic S, Grama A, Pop TL (2022) An update on the effects of vitamin D on the immune system and autoimmune diseases. Int J Mol Sci 23:9784. 10.3390/ijms23179784 36077185 10.3390/ijms23179784 PMC9456003

[18] Holick MF (2007) Vitamin D deficiency. N Engl J Med 357:266–281. 10.1056/nejmra070553 17634462 10.1056/NEJMra070553

[19] Spiro A, Buttriss JL (2014) Vitamin D: an overview of vitamin D status and intake in Europe. Nutr Bull 39:322–350. 10.1111/nbu.12108 25635171 10.1111/nbu.12108 PMC4288313

[20] Stastna D, Drahota J, Lauer M et al (2024) The Czech National MS Registry (ReMuS): data trends in multiple sclerosis patients whose first disease-modifying therapies were initiated from 2013 to 2021. Biomed Pap 168:262–270. 10.5507/bp.2023.015 10.5507/bp.2023.015 37114703

[21] Carter GD, Berry J, Cavalier E et al (2020) Biotin supplementation causes erroneous elevations of results in some commercial serum 25-hydroxyvitamin D (25OHD) assays. J Steroid Biochem Mol Biol 200:105639. 10.1016/j.jsbmb.2020.105639 32084550 10.1016/j.jsbmb.2020.105639

[22] Mansoor F, Kumar V, Kumar S et al (2021) Association between serum vitamin D levels and frequency of relapses in patients with multiple sclerosis. Cureus 13. 10.7759/cureus.14383 10.7759/cureus.14383 PMC8110290 33987049

[23] Fitzgerald KC, Munger KL, Köchert K et al (2015) Association of vitamin D levels with multiple sclerosis activity and progression in patients receiving interferon beta-1b. JAMA Neurol 72:1458–1465. 10.1001/jamaneurol.2015.2742 26458124 10.1001/jamaneurol.2015.2742

[24] Anna AR, Suzan AR, Helmii M (2013) The effect of vitamin D supplementation on inflammatory and hemostatic markers and disease activity in patients with systemic lupus erythematosus: a randomized placebo-controlled trial. J Rheumatol 40:265–272. 10.3899/jrheum.111594 23204220 10.3899/jrheum.111594

[25] Tang J, Shan S, Li F, Yun P (2023) Effects of vitamin D supplementation on autoantibodies and thyroid function in patients with Hashimoto’s thyroiditis: a systematic review and meta-analysis. Medicine. 10.1097/md.0000000000036759 38206745 10.1097/MD.0000000000036759 PMC10754614

